# Intake of Sweets, Snacks and Soft Drinks Predicts Weight Gain in Obese Pregnant Women: Detailed Analysis of the Results of a Randomised Controlled Trial

**DOI:** 10.1371/journal.pone.0133041

**Published:** 2015-07-20

**Authors:** Kristina M. Renault, Emma M. Carlsen, Kirsten Nørgaard, Lisbeth Nilas, Ole Pryds, Niels J. Secher, Sjurdur F. Olsen, Thorhallur I. Halldorsson

**Affiliations:** 1 Department of Obstetrics and Gynaecology, Hvidovre Hospital, University of Copenhagen, Copenhagen, Denmark; 2 Department of Obstetrics and Gynaecology, Odense University Hospital, University of Southern Denmark, Odense, Denmark; 3 Department of Paediatrics, Hvidovre Hospital, University of Copenhagen, Copenhagen, Denmark; 4 Department of Endocrinology, Hvidovre Hospital, University of Copenhagen, Copenhagen, Denmark; 5 The Research Unit Women's and Children's Health, the Juliane Marie Centre, Rigshospitalet, University of Copenhagen, Copenhagen, Denmark; 6 Centre for Foetal Programming, Department of Epidemiology Research, Statens Serum Institut, Copenhagen, Denmark; 7 Department of Nutrition, Harvard School of Public Health, Boston, Massachusetts, United States of America; 8 Faculty of Food Science and Nutrition, University of Iceland, Reykjavik, Iceland; The Chinese University of Hong Kong, HONG KONG

## Abstract

**Background:**

Lifestyle interventions targeting obese pregnant women often result in modest reduction in gestational weight gain, pregnancy complications and related risk factors. Examining adherence to the intervention can, however, provide valuable information on the importance of the different factors targeted.

**Objective:**

To evaluate improvements and relevance of different dietary factors targeted with respect to gestational weight gain in a 3-arm Randomised Controlled Trial (n=342) among obese pregnant women with BMI≥30 kg/m^2^.

**Methods:**

Randomisation 1:1:1 to either hypocaloric Mediterranean type of diet and physical activity intervention (D+PA); physical activity intervention alone (PA); or control (C). Diet was assessed at baseline (weeks 11–14) and endpoint (weeks 36–37) using a validated food frequency questionnaire.

**Results:**

During the intervention women in the D+PA group significantly lowered their intakes of added sugars and saturated fat and increased their protein intake by ~1% of total energy compared to controls. Of these dietary variables only intakes of added sugar appeared to be related to GWG, while no association was observed for saturated fat or protein. Further analyses revealed that foods that contributed to intake of added sugars, including sweets, snacks, cakes, and soft drinks were strongly associated with weight gain, with women consuming sweets ≥2/day having 5.4 kg (95% CI 2.1-8.7) greater weight gain than those with a low (<1wk) intake. The results for soft drinks were more conflicting, as women with high weight gain tended to favour artificially sweetened soft drinks.

**Conclusion:**

In our sample of obese pregnant women, craving for sweets, snacks, and soft drinks strongly predicts GWG. Emphasis on reducing intakes of these foods may be more relevant for limiting gestational weight gain than encouraging strict compliance to more specific diets.

**Trial Registration:**

ClinicalTrials.gov NCT01345149

## Introduction

Obesity is globally a growing threat to women of childbearing age, and high maternal pre-pregnancy weight is a strong risk-factor for pregnancy and delivery complications such as diabetes, hypertension, and preeclampsia [1,2] and for giving birth to a macrosomic neonate [2]. Excessive weight gain in pregnancy is independently associated with macrosomia and adverse pregnancy outcomes [3], and macrosomia is a predictor of childhood and adult obesity [4,5]. The Institute of Medicine (IOM) recommends that obese women limit their gestational weight gain (GWG) to 5–9 kg [6]. In reality few women meet this criteria and mean GWG is often around 10 kg on average [7,8]. Although weight gain above 5 kg is recommended to avoid an increased risk of intrauterine growth restriction, low weight gain (GWG <5kg) has in some studies been associated with reduction in risk for a number of pregnancy complications with no indications of adverse birth outcomes detected [9,10].

Lifestyle interventions have been shown to reduce GWG, and dietary interventions appear to be more effective than interventions based on physical activation alone [11]. However, with the exception of one small (n = 50) and intensive study on obese pregnant women achieving mean reduction in GWG of around 6 kg [12], reduction in GWG as a result of dietary intervention have generally been modest (<2kg) [13–15]. The focus of these previous interventions covered “low glycaemic index (GI) diet” [13–15], Mediterranean diet [7,8], or simple dietary counseling with a brochure [16]. The modest reduction in GWG reported in these studies suggests that failure to comply with the diet may be more important than the type of diet promoted in the intervention. Furthermore, only few studies have evaluated the effect of intervention on specific dietary changes in obese pregnant women [12,16,17].

In line with results from other trials our *Treatment of Obese Pregnant Women (TOP)- study* (n = 425) also resulted in a modest reduction in GWG with median of -1.5 kg among those randomised to physical activity intervention and -2.3 kg among those randomised to physical activity and dietary intervention compared to controls [8]. The objective of this study was to evaluate the impact of the lifestyle interventions on the women’s diet during the course of pregnancy and to examine, what dietary preference may account for the modest reduction in GWG achieved as a result of the intervention.

## Material and Methods

### The TOP-study

The TOP-study [8] was conducted at Hvidovre Hospital, Copenhagen University between April 2009 to March 2012. Enrolment of participants started April 1^st^ 2009 and the last participant was enrolled September 14^th^ 2011. Collection of data in the current study was completed March 14^th^ 2012, the date of delivery for the last participant. Prior to conducting the study a pilot project including 70 obese pregnant women was undertaken [18], and power calculations performed on the basis of that study suggested, that 112 participants were needed per group for being able to detect a 2-kg difference in GWG between groups (α = 0,05 β = 0,80). Expecting some dropouts we planned to include 140 in each group, in total N = 420.

A total of 425 women were enrolled in gestational week 11–14. Inclusion criteria were a pre-pregnancy BMI ≥ 30 kg/m^2^, gestational age < 16weeks, age > 18 years, no disease limiting physical activity, and a normal singleton pregnancy. All women fulfilling these criteria were approached consecutively. Prior to enrolment, all candidates were invited to an initial consultation with a dietician, as this was part of the standard procedure in the department. If they accepted participation in the study, written informed consent was obtained. Participants were encouraged to limit GWG to <5 kg, and were randomised 1:1:1 to (PA+D) physical activity intervention + dietary intervention; (PA) physical activity intervention only; or (C) control. The randomization was non-blinded to the dietician, but blinded to the staff registering GWG and data on the offspring.

*The dietary intervention* was administered by trained dieticians, with follow-up every 2 weeks. Participants were asked to adhere to a hypocaloric diet (5000–7000 kJ) low in saturated fat corresponding to a Mediterranean-style diet in line with Danish national recommendations [19].

The women allocated to *physical activity intervention* (groups PA+D and PA) were immediately after randomization individually advised and encouraged by the dietician to increase physical activity, aiming at a daily step count of 11,000, which corresponds to 150% of the average step count in healthy lean pregnant women [18]. A pedometer was allocated to all subjects in these two intervention groups, but not to controls. The pedometer was worn for seven consecutive days every four weeks. They were reminded through text message in the beginning of each recording period, and the women themselves registered and reported their pedometer output. In week 17, the chart with step counts and weight was returned by 64% in group PA+D and 55% in group PA while the corresponding numbers were 53% and 56% in week 33. There was no difference in the median step counts between the two intervention groups, and in all reporting periods less than 20% of subjects archived 11.000 steps/day.

### Maternal lifestyle and diet

Information on socio-demographic factors, lifestyle and health were recorded at baseline (weeks 11–14), and diet was again recorded at endpoint (weeks 36–37). A self-administered Food Frequency Questionnaire (FFQ) was used covering intakes during the previous 4 weeks [20]. The FFQ has been validated using a 7-day weighted food record and biomarkers as a standard [21]. Assessment of dietary and nutritional intake was done by means of a national food composition database (http://www.foodcomp.dk) using standard recipes and standard portion sizes.

### Outcome assessment

GWG was assessed as the difference between self-reported pre-pregnancy weight recorded at baseline and weight measured at endpoint on an electronic scale (SV-Seca 769), with the woman wearing light indoor clothing and no shoes.

Dietary changes as a result of the intervention were assessed relative to the control group. When evaluating dietary changes we first examined the main energy bearing nutrients.

### Subjects available for analyses

**Fig 1:**
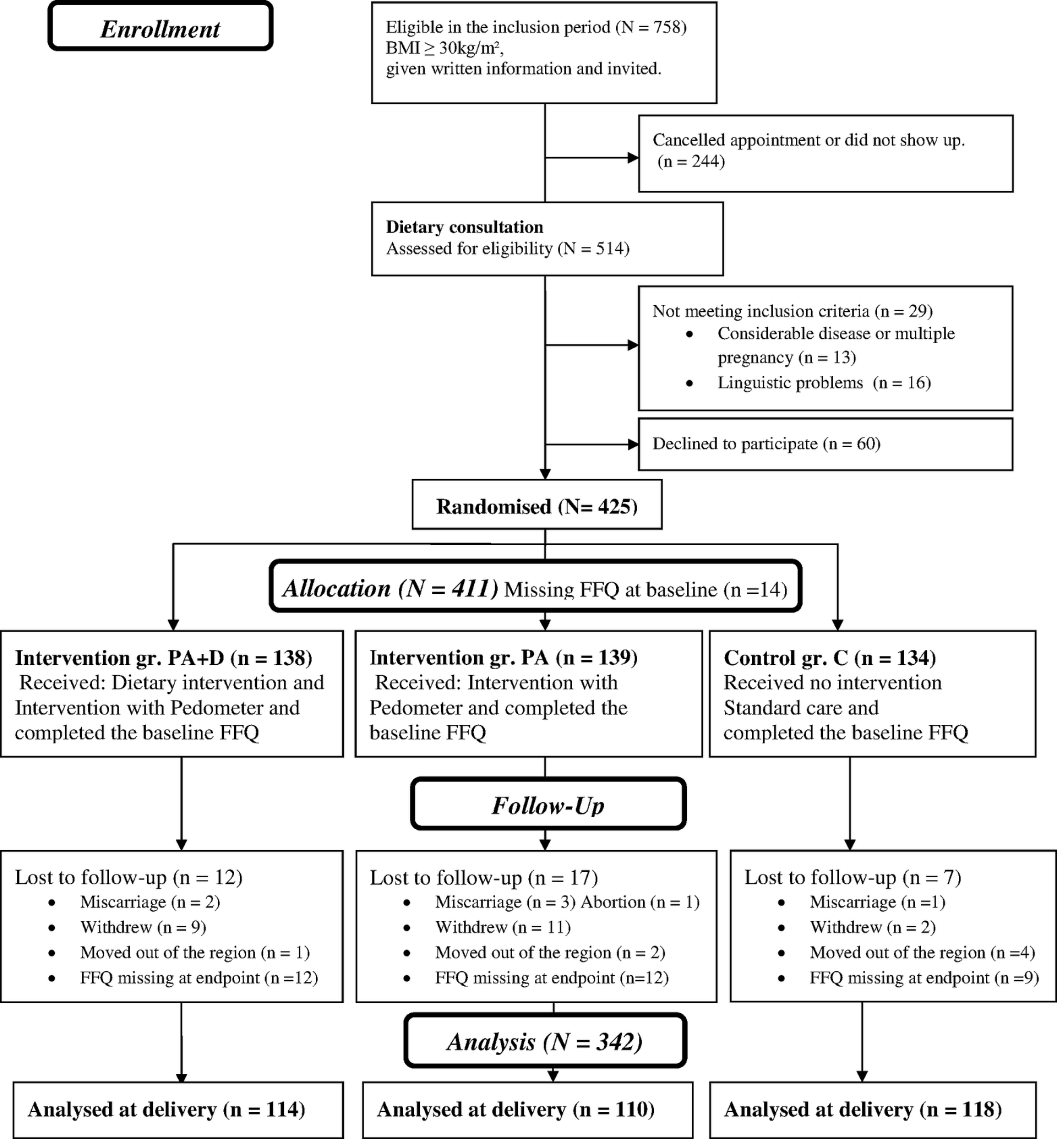
Flow Diagram. The flow diagram describes allocation and inclusion in the TOP-study and in the current study.

Inclusion and attrition rates are presented in Fig 1. Of the 425 women enrolled, 366 (86%) women filled out the first FFQ at baseline and completed the trial. Of these 342 (80%) also filled out the FFQ at endpoint.

### Ethics

This study was conducted according to the guidelines laid down in the Declaration of Helsinki, and all procedures were approved by the Ethics Committee for the Capital Region of Denmark (January 26th 2009) (ID Number H-D-2008-119) (S3, S4 and S5 Files). Written informed consent was obtained from all subjects. The TOP-study is registered at ClinicalTrials.gov (ID Number NCT01345149). Unfortunately when we wrote the protocol in 2008 and started inclusion of participants in 2009, we were unaware of the new ICMJE rules, and the study was not registered at ClinicalTrials.gov before enrolment of participants was started. After being aware of the rules in the inclusion period, we registered the study immediately. This was further delayed because of some technical difficulties when submitting. But the protocol was approved by the Ethics Committee before inclusion of participants was started; and the study has been conducted without any deviations from the detailed protocol. The authors confirm that all ongoing and related trials for this intervention are registered.

### Statistical analyses

When examining the effect of the dietary intervention we restricted our analyses to subjects who filled out the FFQ at both baseline and endpoint (n = 342). For describing the subject’s characteristics at baseline we used the mean and standard deviation for continuous measures and the t-test to test for differences between groups. For skewed variables the Wilcoxon rank sum test was used to test for differences between groups. Percentages were used to describe dichotomous measures and the χ2-square test was used to test for differences between groups.

The effect of the intervention in terms of dietary changes between baseline (weeks 11–14) and endpoint (weeks 36–37) were evaluated as each subject’s change (Δ) for dietary variable *i*: Δ*i* = endpoint (i)–baseline (i). As women may change their dietary habits during the course of pregnancy, dietary changes as a result of the intervention (as per protocol analyses) were assessed relative to the control group. When evaluating dietary changes we first examined the main energy bearing nutrients. These changes are better suited to capture the accumulated change in the diet, as opposed to examining changes in individual foods or food groups

The influence of nutrients affected by the intervention were then examined in relation to GWG using linear regression analyses. We used all subjects that filled out the FFQ at baseline and completed the trial (n = 366). In these analyses we used substitution models [22] examining: *i*) for carbohydrate a 1:1 energy substitution of added sugars for other carbohydrates; *ii*) for fats, a 1:1 energy substitution of one type of fat for another, such as saturated fat replaced by either poly- or mono-unsaturated fat; *iii*) and for proteins: a 1:1 energy substitution of replacing proteins by carbohydrates. The relevance of alternative substitutions was also explored in stability analyses.

The association between individual foods related to these nutrients and GWG were also examined, when appropriate, for further clarification. In these regression analyses analyses we selected, *a priori*, and adjusted for the following set of covariates: total energy intake, maternal age, parity, smoking during pregnancy, pre-pregnancy body mass index, and intervention group. The statistical package SAS, version 9,2, Cary, NC was used.

## Results

The flow diagram (Fig 1) describes allocation and inclusion in the TOP-study and in the current study. Comparing those who filled out both FFQ’s (n = 342) with those who dropped out of the study (n = 83) respectively, the mean birth weight was 3656 compared to 3259g (p<0.001), maternal age was 31.4 compared to 30.0 years (p = 0.005), pre-pregnancy BMI was 33.8 compared to 35.4 kg/m2 (p = 0.001) and prevalence of smoking in pregnancy was 8% compared to 4% (p = 0.24). Despite these differences, the randomization appears to have been retained as non-significant differences in baseline characteristics were observed between then controls and the two intervention groups among the 342 participants included in this study (Table 1). The mean GWG for the subjects in Table 1 population (n = 342) was 10 kg. The prevalence of women with low (<5kg) normal (5-9kg) and excessive weight gain (>9kg) was 21%, 26% and 53%, respectively.

**Table 1:** Maternal characteristics and dietary intakes of selected macronutrients at baseline among participants reporting their diet at baseline (wk 11–14) and endpoint (wk 36–37) in the TOP-Study (n = 342).

	Control	Physical Activity	Physical Activity
	(n = 118)	(n = 110)^1^	+ Diet (n = 114)^1^
Age (SD) (years)	31.4 (4.2)	31.3 (4.7)	31.5 (4.0)
Pre-preg. BMI (kg/m^2^)	33.4 (3.3)	33.8 (4.0)	34.1 (4.0)
Parity (%)	46	43	43
Smoking (%)	8	6	8
Energy (MJ/day)	8.1 (3.5)	7.9 (2.6)	8.0 (2.1)
Protein (%E)	16.8 (2.7)	17.2 (2.3)	17.0 (2.6)
from animal sources	9.9 (3.0)	10.4 (2.7)	10.1 (2.9)
from plant sources	6.9 (2.3)	6.8 (1.8)	7.0 (2.1)
Carbohydrates (%E)	51.1 (6.7)	50.1 (6.1)	50.6 (5.7)
Added sugar (%E)	7.3 (5.2)	6.8 (4.7)	6.6 (4.5)
from foods	5.7 (3.8)	5.1 (2.6)	5.5 (3.7)
from soft drinks	1.6 (3.8)	1.6 (4.2)	1.2 (2.7)
Fat (%E)	32.1 (6.2)	32.7 (6.1)	32.4 (5.9)
saturated	12.4 (3.1)	12.9 (3.5)	13.1 (3.2)
monounsaturated	10.7 (2.4)	10.9 (2.3)	10.1 (2.1)
polyunsaturated	5.1 (1.1)	5.2 (1.2)	4.9 (0.8)

Abbreviations: SD: Standard deviation; %E: % of total energy intake.

^1^Maternal characteristics and dietary intakes in the intervention groups were not significantly different (p>0.05) from the control group for all variables reported in the table (Chi-square test for parity and smoking, Wilcoxon rank sum test for the added sugar variables, otherwise t-test).

### Changes in energy bearing nutrients and GWG

Dietary changes between baseline and endpoint for the 342 subjects, who reported their diet at baseline and endpoint, are shown in Table 2. No significant changes in dietary intake were observed in the PA group compared to controls for the 12 dietary variables tested. For the PA+D group, there was, however, a shift towards healthier diet, with significant increase in intakes of protein and polyunsaturated fatty acids and decreased intakes of added sugars and saturated fat compared to controls. The increase in protein intake was due to higher intakes of animal protein while the decrease in intake of added sugars was driven by a decrease in both added sugars from soft drinks and foods. However, the shift in diet in the PA+D group was in all cases modest or around ~1% of energy intake. Energy intake, as assessed by the FFQ, was not reduced in the two intervention groups compared to controls. Coinciding with the shift towards slightly healthier diet, women in the PA+D group had significantly lower relative risk (RR) of excessive weight gain compared to controls [0.73 (95%CI: 0.57–0.94)]. At the same time the relative risk of having GWG below 5kg was slightly increased [1.33 (95%CI: 0.80–2.21)] among women in the PA+D group compared to controls. In this context, it is worth noting that in this study we found no indication of adverse pregnancy outcomes including intrauterine growth restriction among those women who had GWG below 5kg. Non-significant difference in GWG was observed for the PA group compared to controls (Table 2).

**Table 2:** Relative changes in dietary intake between baseline and endpoint among subjects reporting their diet at both time points (N = 342). The relative risk of having either low or excessive weight gain as defined by the institute of medicine^1^ is also shown for each group.

	Control	Physical Activity	Physical Activity
	(n = 118)	(n = 110)	+ Diet (n = 114)
*Changes in dietary intake*		Δ^2^ (95% CI)	Δ^2^ (95% CI)
Energy (MJ/day)	Reference	-0.0 (-0.7, 0.7)	0.1 (-0.5, 0.8)
Protein (%E)	Reference	0.3 (-0.4, 1.0)	**1.0 (0.3, 1.7)**
Animal protein	Reference	0.0 (-0.8, 0.9)	**1.1 (0.2, 1.9)**
Plant protein	Reference	0.3 (-0.3, 0.8)	0.0 (-0.6, 0.5)
Carbohydrates (%E)	Reference	-0.1 (-1.7, 1.8)	-0.9 (-2.6, 0.8)
added sugar	Reference	0.1 (-1.2, 1.5)	**-1.3 (-2.6, -0.0)**
form foods	Reference	0.6 (-0.3, 1.5)	-0.8 (-1.7, 0.1)
from soft drinks	Reference	-0.4 (-1.5, 0.6)	-0.5 (-1.5, 0.4)
Fat (%E)	Reference	-0.3 (-1.9, 1.3)	-0.4 (-2.0, 1.2)
saturated	Reference	-0.4 (-1.2, 0.5)	**-0.8 (-1.6, -0.0)**
Monounsaturated	Reference	0.1 (-0.6, 0.7)	0.2 (-0.5, 0.8)
Polyunsaturated	Reference	0.0 (-0.3, 0.4)	**0.4 (0.1, 0.7)**
*Gestational weight gain*		RR (95% CI)	RR (95% CI)
low weight gain (<5kg)	Reference	1.24 (0.73, 2.09)	1.33 (0.80, 2.21)
excessive (>9kg)	Reference	0.86 (0.68, 1.08)	0.73 (0.57, 0.94)

Abbreviations: CI: Confidence interval; RR relative risk

^1^ Institute of Medicine and National Research Council. Weight gain during pregnancy: re-examining the guidelines. Washington (DC): National Academy Press; 2009.

^2^mean difference (Δ = endpoint—baseline) compared to the reference group.

There were some indications, that those women in the PA group who were relatively physically active, as assessed by the pedometer output in week 17, changed their diet in a similar direction as the women in the PA+D group (Table 3). However, given the reduced number of women when stratifying by activity level in the PA group, it was difficult to assess if these changes in diet in the PA group could have accounted for the non-significant reduction in excessive GWG.

**Table 3:** Dietary changes in the physically activity intervention groups stratified by median pedometer output in week 17 of gestation. Corresponding changes in GWG are also presented.

	Control	Physical Activity	Physical Activity	Physical Activity
		*low activity* ^*1*^	*high activity* ^*1*^	+ Diet
*Endpoint—baseline*		Δ^2^ (95% CI)	Δ^2^ (95% CI)	Δ^2^ (95% CI)
No.	118	60	47	114
Prot (%E)	Reference	-0.1 (-1.3, 0.7)	1.0 (0.1, 2.0)	1.1 (0.4, 1.8)
Added sugar (%E)	Reference	1.2 (-0.3, 2,8)	-1.2 (-3.0, 0.5)	-1.3 (-2.6, -0.0)
Saturated fat (%E)	Reference	-0.3 (-1.3, 0.7)	-0.5 (-1.7, 0.6)	-0.8 (-1.6, -0.0)
Poly unsaturated (%E)	Reference	0.1 (-0.3, 0.4)	-0.0 (-0.4, 0.4)	0.4 (0.1, 0.7)
*Gestational weight gain*				
low weight gain (<5kg)	Reference	1.22 (0.66, 2.26)	1.20 (0.61, 2.34)	1.33 (0.80, 2.21)
excessive (>9kg)	Reference	0.87 (0.66, 1.15)	0.84 (0.61, 1.15)	0.73 (0.57, 0.94)

^1^Low activity is defined as pedometer output below the median value (8725 steps/day) or missing on pedometer output (n = 13). High activity is defined as pedometer output above the median

^2^ mean difference (Δ) and 95% confidence interval (95%CI). Dietary variables are adjusted for baseline BMI, maternal age, smoking, parity, and baseline energy intake

### Dietary predictors of GWG

Associations between baseline intake of those dietary variables that were significantly affected by the intervention and GWG are shown in Table 4 for all 366 subjects who filled out the FFQ at baseline. In short, non-significant associations were seen for protein (including animal and plant protein), intake of total added sugar, saturated and polyunsaturated fats. However intake of added sugars form foods was positively associated (p for trend = 0.02) with GWG. The observed difference was 2.8 kg (95% confidence interval: 0.8–4.8) when comparing the women with the highest to lowest quartile of intake at baseline. On the other hand intake of added sugar from soft drinks (carbonated and non-carbonated) showed an inverse non-significant trend (p for trend = 0.13)

**Table 4:** Association^1^ between intake of selected macronutrients at baseline and gestational weight gain (n = 366). Associations are explored for those macronutrients that were affected by the dietary intervention as presented in Table 2.

	Δkg^2^ (95% CI)		Δ kg ^2^ (95% CI)		Δ kg ^2^ (95% CI)
***Protein*** *(median*, *%E)* ^*3*^	Total Protein	(median, %E)	from Animal sources	(median, %E)	from plant sources
Quartile 1 (14.0)	Reference		Reference		reference
Quartile 2 (16.2)	0.4 (-1.5, 2.2)	(7.1)	-0.1 (-1.9, 1.8)	(4.8)	0.9 (-0.9, 2.7)
Quartile 3 (17.8)	0.9 (-1.0, 2.7)	(9.2)	0.4 (-1.4, 2.2)	(5.8)	1.4 (-0.4, 3.3)
Quartile 4 (20.0)	0.0 (-1.9, 1.9)	(11.0)	0.0 (-1.9, 1.9)	(7.0)	-0.2 (2.1, 1.7)
P for trend^4^	0.86	(13.6)	0.87	(9.1)	0.99
***Added sugar*** *(median*, *%E)* ^*5*^	Total Added sugar		from foods		from soft drinks
Quartile 1 (3.0)	Reference	(2.6)	Reference	(0.0)	reference
Quartile 2 (4.8)	1.6 (-0.3, 3.4)	(4.1)	**2.1 (0.2, 3.9)**	(0.2)	-0.3 (-2.1, 1.5)
Quartile 3 (6.9)	0.8 (-1.1, 2.6)	(5.5)	**1.3 (-0.5, 3.2)**	(0.8)	-1.1 (-2.9, 0.8)
Quartile 4 (10.1)	0.4 (-1.7, 2.5)	(8.8)	**2.8 (0.8, 4.8)**	(2.8)	-1.3 (-3.2, 0.6)
P for trend^4^	0.82		**0.02**		0.13
***Fats*** *(median*, *%E)* ^***6***^	Saturated fat		Polyunsaturated fat		
Quartile 1 (9.2)	Reference	(4.0)	Reference		
Quartile 2 (11.6)	-0.5 (-2.3, 1.3)	(4.7)	-1.0 (-2.8, 0.8)		
Quartile 3 (13.5)	-0.2 (-2.0, 1.5)	(5.3)	1.3 (-0.5, 3.1)		
Quartile 4 (16.8)	-0.3 (-2.2, 1.6)	(6.1)	-0.4 (-2.2, 1.4)		
P for trend^4^	0.83		0.75		

Abbreviations: Q: quintile. CI: Confidence interval.

^1^ The associations presented in the table were explored for each nutrient separately. All associations are adjusted for energy intake, maternal age, smoking during pregnancy, parity (0/1) and pre-pregnancy body mass index and intervention group

^2^ mean change in GWG compared with the reference (in kg)

^3^ 1:1 energy substitution of increasing protein intake on the expense of for carbohydrates.

^4^ t test (ordinal values entered)

^5^ 1:1 energy substitution of increasing intakes of added sugars on the expense of other carbohydrates.

^6^ 1:1 energy substitution of increasing intake of saturated fats on the expense of mono- or polyunsaturated fats; increasing intake of polyunsaturated fat on the expense of saturated or mono-unsaturated fat.

To clarify the conflicting association for different sources of added sugars their main food sources were explored further in relation to GWG (Table 5, left panel). These analyses revealed that baseline (week 11–14) intakes of sweets (e.g. chocolates, caramels, liquorice, gummy and jelly sweets), snacks (e.g. chips, salted peanuts, popcorn), cakes and ice cream were relatively strong predictors of GWG. For example women consuming sweets two times a day or more at baseline had on average 5.4 kg (95%CI: 2.1–8.7) greater GWG compared to women consuming sweets less than once a week. This mean increase in GWG corresponded to a relative risk of 1.84 (95%CI 1.14–2.96) of having excessive GWG (>9kg). More modest, but significant associations were observed for cakes and ice cream and snacks. With respect to soft drinks we observed, at baseline, an inverse association with GWG for sugar-sweetened carbonated soft drinks (p for trend = 0.02) with women consuming one or more drinks per day having on average 3.1 lower GWG (95%CI: -0.5, 6.0) compared to women with no consumption. On the other hand women reporting consuming artificially sweetened carbonated soft drinks at baseline had on average 2.0 kg (95%CI: -0.2, 4.2) higher GWG compared to women with no consumption, although the overall trend was non-significant. At endpoint (Table 5, right panel), near identical associations between consumption of sweets and GWG were observed while the associations for snacks, cakes and ice cream were attenuated. At endpoint, the association between artificially sweetened carbonated soft drinks also became slightly stronger and significant.

**Table 5:** Associations^1^ between self-reported intake at baseline and endpoint of sweets snacks, cakes and soft drinks with gestational weight gain. Associations for both relative risk (RR) of excessive weight gain (>9kg) and mean increase in GWG compared to the reference category (Δ) are presented.

	Baseline (n = 366)			Endpoint (n = 347)		
	Mean change	Excessive weight gain (>9kg)		Mean change	Excessive weight gain (>9kg)	
*Sweets*	Δkg (95% CI)	Cases/n	RR (95% CI)	Δkg (95% CI)	Cases/n	RR (95% CI)
<1/wk	Reference	21/54	1.00	Reference	21/51	1.00
1–<3/wk	2.6 (0.7, 4.6)	62/131	1.28 (0.89, 1.84)	0.4 (-1.6, 2.4)	42/108	0.99 (0.66, 1.47)
3–<7/wk	3.6 (1.6, 5.6)	63/109	1.52 (1.06, 2.22)	3.3 (1.3, 5.3)	85/133	1.51 (1.06, 2.15)
1/d	4.5 (2.1, 6.9)	35/54	1.71 (1.17, 2.59)	3.2 (0.8, 5.7)	31/49	1.50 (1.01, 2.23)
≥2/d	5.4 (2.1, 8.7)	12/18	1.84 (1.14, 2.96)	5.6 (0.5, 10.8)	4/6	1.66 (0.83, 3.30)
P for trend^2^	0.0009		0.0006	<0.0001		0.001
*Snacks*						
<1/wk	Reference	48/110	Reference	Reference	59/115	1.00
1–<2/wk	1.8 (0.1, 3.6)	50/90	1.28 (0.97, 1.70)	0.1 (-1.5, 1.8)	54/103	1.02 (0.79, 1.32)
2–<3/wk	1.9 (0.1, 3.7)	42/76	1.26 (0.96, 1.67)	-0.5 (-2.5, 1.4)	29/58	0.95 (0.70, 1.30)
≥3/wk	2.4 (0.7, 4.2)	53/90	1.33 (1.02, 1.75)	0.3 (-1.5, 2.2)	41/71	1.06 (0.81, 1.39)
P for trend^2^	0.01		0.04	0.91		0.81
*Cakes*						
<1wk	Reference	13/36	Reference	Reference	13/35	1.00
1–<2/wk	2.1 (-0.5, 4.7)	35/59	1.57 (0.98, 2.51)	1.0 (-1.6, 3.7)	31/51	1.60 (0.99, 2.59)
2–<3/wk	1.6 (-0.9, 4.2)	29/65	1.26 (0.76, 2.07)	1.7 (-0.9, 4.3)	31/63	1.33 (0.81, 2.18)
3–<5/wk	2.5 (0.1, 4.9)	57/106	1.47 (0.93, 2.32)	1.0 (-1.5, 3.4)	51/95	1.33 (0.84, 2.18)
≥5/wk	3.0 (0.6, 5.5)	59/100	1.56 (0.99, 2.47)	2.1 (-0.4, 4.5)	57/103	1.39 (0.88, 2.20)
P for trend^2^	0.04		0.02	0.18		0.59
*Sugar sweetened carbonated soft drinks*						
0	Reference	77/133	Reference	reference	66/125	1.00
<1/day	-1,4 (-2.8, -0.05)	105/211	0.79 (0.65, 0.96)	-0.0 (-1.4, 1.4)	106/196	0.96 (0.77, 1.19)
≥1/day	-3.1 (-6.0, -0.05)	10/21	0.62 (0.36, 1.05)	-1.4 (-4.2, 1.5)	11/25	0.71 (0.44, 1.14)
P for trend^2^	0.02		0.001	0.52		0.23
*Artificially Sweetened carbonated soft drinks*						
0	Reference	83/156	Reference	reference	64/133	1.00
<1/day	-0.5 (-1.9, 0.9)	79/170	0.87 (0.70, 1.07)	0.9 (-0.5, 2.3)	86/162	1.08 (0.87, 1.38)
≥1/day	2.0 (-0.2 4.2)	30/40	1.50 (1.17 1.92)	2.5 (0.5, 4.5)	33/52	1.43 (1.10, 1.86)
P for trend^2^	0.36		0.16	0.02		0.02

Abreviations: RR: Relative Risk

^1^ The associations presented in the table were explored for each food separately. All associations are adjusted for energy intake, maternal age, smoking during pregnancy, parity (0/1), and pre-pregnancy body mass index and intervention group

^2^ t test (ordinal values entered)

Concerning the results presented in Table 5 the covariates included in our analyses had modest impact on our estimates when compared to unadjusted analyses (data not shown). As an example the unadjusted increase in GWG for baseline intake of sweet comparing the highest (≥2/d) to lowest (<1/wk) category was 4.8kg (95%CI: 1.4–8.2), while the fully adjusted estimate presented in Table 5 was 5.4kg (95CI: 2.1, 8.7). In additional analyses, we also examined the stability of our findings by stratifying by parity (primi- versus multipara). In those analysed near identical associations were observed for primi- and multiparous women (data not shown).

Given the consistency and strength observed for the association between sweets and GWG, the correlation between sweets and intake of selected macro- and micronutrients at baseline was examined further (Table 6). Going from the lowest (<1/week) to the highest (≥2/day) category of sweets, total energy intake increased by around 27% (6.5 to 8.9 MJ/day), and intakes of added sugar approximately doubled (5.4 to 11.1% of total energy). The intake of protein and folic acid was also markedly reduced, while saturated fat intake increased. Intakes of sweets, cakes, or added sugars at baseline or at endpoint did not correlate with the level of physical activity measured by pedometer in weeks 17 and 33 (Spearman rho< 0.1, P>0.25).

**Table 6:** Characteristics of study participants and mean macronutrient intake across categories of self-reported consumption of sweets at baseline (n = 366).

	<1/wk	1–<3/wk	3–<7/wk	1/d	≥2/d	P^1^
N	54	131	109	54	18	
Age (SD) (years)	30.9 (4.0)	31.2 (4.1)	31.4 (4.2)	31.6 (5.1)	30.8 (5.2)	0.59
BMI (SD) (kg/m2)	33.5 (3.7)	33.9 (3.6)	34.1 (4.4)	34.5 (4.8)	34.5 (4.5)	0.18
Smoking	2%	7%	6%	9%	28%	0.009
Primipara mothers	37%	45%	47%	50%	44%	0.70
Sugar sweetened soft drinks (≥1d)	4%	9%	7%	4%	6%	0.49
Artificially sweetened soft drinks (≥1d)	2%	10%	15%	24%	13%	0.005
*Nutrient intake*						
Energy (MJ/day)	6.5 (2.0)	7,6 (2.2)	8.4 (3.3)	9.2 (2.9)	8.9 (3.3)	<0.0001
Protein (%E)	17.6 (2.6)	17.6 (2.5)	16.5 (2.4)	16.8 (2.5)	15.3 (2.7)	<0.0001
Carbohydrates (%E)	51.1 (7.0)	50.7 (6.1)	52.3 (6.0)	49.9 (5.6)	50.9 (8.9)	0.94
added sugar	5.4 (4.1)	6.0 (3.9)	7.4 (5.5)	7.8 (2.6)	11.1 (8.1)	<0.0001
fibres (g/day)	23.8 (7.8)	22.5 (6.0)	22.4 (5.5)	19.9 (4.9)	17.6 (5.9)	<0.0001
Fat (%E)	31.5 (7.2)	32.1 (5.6)	31.8 (5.6)	34.1 (5.9)	34.9 (7.5)	0.01
saturated	12.5 (3.9)	12.7 (3.0)	12.5 (3.1)	13.5 (3.1)	14.2 (4.5)	0.04
Monounsaturated	10.4 (2.3)	10.7 (2.3)	10.5 (2.0)	11.3 (2.1)	11.3 (2.9)	0.05
Polyunsaturated	5.0 (0.9)	5.1 (1.1)	5.1 (1.0)	5.1 (1.0)	4.8 (1.3)	0.78
Vitamin C (mg/day)	128 (77)	124 (58)	134 (94)	102 (42)	103 (83)	0.10
Folic acid (μg/day)	315 (105)	296 (70)	294 (73)	282 (87)	244 (75)	0.003

^1^ t test (ordinal values entered). For parity, soft drinks and smoking where Chi-square test is used

## Discussion

The aim of this study was to evaluate the effect of lifestyle interventions on dietary habits in obese pregnant women, and to identify dietary factors that may have accounted for the modest reduction observed in GWG in our full randomized controlled trial [8]. These analyses were conducted in a reduced set of participants (n = 342 or 80% of those enrolled) who reported their diet at baseline and endpoint. Despite reduction in numbers compared to the full trial, the randomisation was still retained and significant effects on both dietary habits and GWG were observed in the group randomised to both dietary and physical activity intervention, but not in the group randomised to physical activity alone. These results suggest that the modest dietary changes observed may have accounted for the reduction in GWG observed. At the same time the results also highlight poor compliance to the dietary intervention as energy intake, as assessed by the FFQ, was not reduced compared to controls, and favourable changes in diet including reduction in added sugars and saturated fats were modest.

Based on these results it was examined further, in a non-randomised setting, whether any of the dietary factors that were affected by the intervention could predict GWG. As no effect on energy intake was observed of the intervention, it was examined whether 1:1 energy substitution of protein for carbohydrates, added sugar for other carbohydrate sources, or substituting one type of fat for another at baseline were related to GWG. In these models non-significant associations with GWG were observed in all cases. Overall, the association between baseline intake of added sugar was not significantly associated with GWG. However when examining intake of added sugars from foods and soft drinks separately, the two sources of added sugar were in opposite direction with added sugar from foods at baseline being significantly positively associated with GWG.

The amount of energy from added sugar in our obese population was considerably lower than what has been reported among women aged 25–34 in national surveys (7% versus 11% of energy) [23], and one could argue that it may not be sufficiently high to be stronger determinant of weight gain compared to other nutrients [24]. However, obese women are known to systematically underestimate their intake [25]. Furthermore, quantification of added sugar is relatively complex as the amount added to comparable food items may vary substantially. Examining the main food sources of added sugars on a frequency level avoided some of those assumptions. In those analyses frequent consumption at baseline of sweets and desserts, snacks and particularly sweets more strongly predicted GWG than combined intake of added sugar from those sources.

Our results on soft drinks were conflicting, but not entirely unexpected. As opposed to other food sources the choice of soft drinks allows selection between sugar-sweetened or artificially sweetened drinks, a choice that is more restrictive when it comes to solid foods. As a result women concerned about their weight or being on a positive trajectory often chose artificial sweetened alternatives [26]. This was clearly observed in our study (Table 5), where intake of sweets was associated with more frequent consumption of artificially sweetened soft drinks, but not of sugar-sweetened soft drinks. Although we acknowledge that the association observed between sweets and GWG may reflect other attributes of unhealthy diet, the results of our study suggests that cutting down on non-nutritive foods may be the most logical starting point to avoid excessive weight gain.

### Results of the present study in relation to other studies

In a small randomized controlled trial of 50 obese Caucasian pregnant women [12] with randomization to intensive dietary advice and follow-up, GWG was restricted to 6.6 kg vs. 13.3 kg in the control group. Dietary registrations using 7-day weighted food records at inclusion in gestational weeks 15±3, 27 and 36 revealed a significant decreased intake of energy from fat. The protein intake also increased significantly. Dietary registrations in week 27 were used for correcting advice, which might be some of the explanation for the very high weight restriction and compliance to the diet.

In an intervention study on cholesterol-lowering diet [7] among 290 normal-weight healthy women, GWG in week 30 was only reduced by around 0.6 kg compared to controls. Dietary 4- or 6-day weighted food records were performed during three periods and showed that the intervention group consumed fewer calories, less saturated and total fat, and cholesterol, but more carbohydrates. In a recently published pilot study of a complex randomized trial of dietary and physical activity intervention in 183 obese pregnant women in which behavioural changes were assessed [17], there was no significant reduction in GWG either. Still, assessment of the diet by repeated triple pass 24-hour recall data at baseline and in week 28 revealed that total energy intake, GL and saturated fat were significantly lower in the intervention group, and the proportion of the energy derived from protein was higher. Intervention [14,15] and observational studies [27,28] examining the role of GI or GL on GWG have also yielded conflicting results [27,28]. Still, a large observational study of around of 47000 pregnant women [28] found a positive association between GWG and GL in normal and overweight, but not in obese women. This is partly in accordance with the current study.

In general, the changes in dietary intake and GWG have been modest in most interventional studies, which most likely relates to poor compliance. The effect among those complying with the intervention is rarely reported making it difficult to evaluate the potential benefits of the diet promoted. Based on the results from the above-mentioned studies, we speculate that compliance to the diet allocated seems more important than type of assigned diet, and motivating women to shift their diet and comply with Mediterranean, low GI, or other types of healthy diet is difficult to achieve in practice. Changing dietary habits is a complex task, and not only the composition of macronutrients, but also the food structure and processing should be taken into account in the evaluation of a diet [29]. Understanding how such factors affect energy balance and pathways involved in hunger, satiety, absorption, and metabolism is currently not well understood [29].

### The strength and weaknesses

The strength of this study is the high rate of completers (81%) and that diet was recorded using a validated FFQ at baseline in the first trimester and again late in pregnancy, giving the possibility to evaluate dietary changes in the participants. One limitation, however, of using a FFQ is that it is not precise in terms of estimating absolute intakes on an individual basis. As a result, the absolute changes in nutrient intake observed in this study are modest, and we suspect that larger changes might have been observed between groups if we had relied on one or repeated 24-hour dietary recall or records. Compared to dietary recall or records, the use of FFQ is more appropriate for capturing average intakes over long periods and more importantly for ranking individuals in terms of high and low intakes, which is important when examining associations with outcomes such as GWG. As with all studies on overweight or obese subjects, underreporting of unhealthy dietary intake is to be expected [25], which may even have attenuated the association between diet and GWG. Finally as in all observational settings, the role of unmeasured confounder(s) cannot be excluded for our results the association between maternal diet and GWG.

## Conclusion and Implications

In this randomised controlled study of obese pregnant women, the dietary intervention resulted in significant but modest improvements in intakes of added sugar, saturated fat, and protein. These changes in diet were also accompanied with decreased relative risk of excessive gestational weight gain, while non-significant effects on diet and GWG were observed in the group randomized to physical activity alone. Examining the dietary changes further suggested, that foods with high content of added sugars relatively strongly predicted GWG, while the changes in protein or saturated fat appeared to be of little importance. For limiting GWG, our results indicate that for obese pregnant women emphasis on reducing intakes of sweets, snacks, and soft drinks may be relatively more important than encouraging strict compliance to specific types of diets. The underlying craving for sweets in study participants may be difficult to modify, but should be considered in future investigations.

## Supporting Information

S1 File Consort Checklist. (DOCX)

S2 File Trend Checklist. (PDF)

S3 File Protocol H-D-2008-119-28.12.08. (DOC)

S4 File Protocol H-D-2008-119 English version. (DOC)

S5 File Approval Ethics Committee H-D-2008-119. (PDF)
