# Supplementary material for: Intake of Sweets, Snacks and Soft Drinks Predicts Weight Gain in Obese Pregnant Women: Detailed Analysis of the Results of a Randomised Controlled Trial
Source: PLoS One. 2015 Jul 20;10(7):e0133041. doi: 10.1371/journal.pone.0133041 (PMC4507874; doi:10.1371/journal.pone.0133041)
Supplement: S3 File — (DOC) [file pone.0133041.s003.doc]

**Effekt af livsstilsintervention i form af diæt og motion samt**

**belysning af kostvaners og biomakørers betydning for graviditets- og fødselskomplikationer hos overvægtige gravide**

Projektgruppe: Afdelingslæge Kristina Renault ^1^

Overlæge Kirsten Riis Andreasen ^1^

Overlæge, dr.med. Lisbeth Nilas ^1^

Professor, Niels Jørgen Secher ^1^

Overlæge Maiken Lundstrøm ^1^

Overlæge,dr.med. Kirsten Nørgaard ^2^

Klinisk Diætist, cand. Scient. Anette Martinsen ^2^

Seniorforsker professor dr.med, Phd. Sjurdur F. Olsen ^3^

1: Gynækologisk Obstetrisk afdeling

2: Endokrinologisk afdeling

Hvidovre Hospital

Kettegårds Allé 30

2650 Hvidovre

3: Afdeling for Epidemiologisk Forskning

Statens Serum Institut,

Artillerivej 5

2300 København S

Der planlægges følgende projekter:

**A. Randomiseret interventionsstudium: Effekt af fysisk aktivitet målt ved anvendelse af pedometer, samt intensiveret diæt-intervention hos overvægtige gravide med BMI≥30 kg/m².**

Et randomiseret interventionsstudium med sammenligning af standard kostvejlednings regime, overfor rekommandation af øget fysisk aktivitet monitoreret ved pedometer samt med og uden intensiveret diætvejledning i anvendelse af middelhavskost. Intensiveret diætvejledningen foretages af diætist med opfølgning hver anden uge, med skiftevis personligt fremmøde og telefon-konsultation.

**FORMÅL:**

- At belyse interventionens effekt på motion, kost, vægtøgning, glukosebelastning, lipidprofil og kliniske parametre ved barnet.
- At belyse faktorer der influerer på deltagelse og kompliance ved livsstilsintervention.

**B.** **Spørgeskemaundersøgelse: Belysning af kostvaner/kostændringer hos overvægtige gravide med BMI≥30 kg/m², med hhv. høj og lav vægtøgning i graviditeten:**

Detaljeret kostanamnese (360 spørgsmål) optages først og sidst i graviditeten ved hjælp af spørgeskema anvendt og valideret i undersøgelsen: ”Bedre sundhed for mor og barn.”

**FORMÅL:**

- At analysere betydningen af ændringer i kostens delelementer for maternel vægtøgning, fostervægt, graviditetslængde og graviditetskomplikationer.

**BAGGRUND:**

Graviditet og overvægt

Overvægt er et stigende problem globalt, og i Danmark er forekomsten af 30-årige svært overvægtige kvinder (BMI **≥** 30 kg/m^2^) steget fra 3.1 til 7.8 % over en 10-årig periode. Denne stigning ser ud til at fortsætte (1).

Overvægtige gravide har en øget risiko for forekomst af en lang række komplikationer i graviditeten såsom gestationel diabetes mellitus (GDM) (2), hypertension (3) samt præeklampsi (4). Et højt BMI øger risikoen for, at fødslen igangsættes (5), for kejsersnit (6) og for operative komplikationer (7). Børnene har en større risiko for medfødte misdannelser, specielt neuralrørsdefekter (8), makrosomi (9) og for intrauterin død (10).

Gravide, som får GDM, har en stor risiko for at udvikle diabetes type 2 senere i livet.

Graviditet og vægtstigning

Sammenhængen mellem vægtstigning og komplikationer i graviditeten er dårligt belyst. I et dansk kohortestudie af overvægtige (BMI **≥** 30 kg/m^2^) kvinder fandtes en sammenhæng mellem vægtstigning og hyppigheden af hypertension, kejsersnit, igangsætning og vækstretardering af fosteret. Der var færrest komplikationer og ingen forekomst af vækstretardering ved en maternel vægtstigning < 5 kg. (11). To store kohortestudier fra 2007, hhv. et svensk studie af 100.000 gravide (12) og et amerikansk studie af 120.000 gravide (13) fandt tilsvarende resultater.

Fra Ernæringsrådet anbefales, at svært overvægtige tager 6-8 kg på i løbet af graviditeten (14). En enkelt dansk interventionsundersøgelse (15), hvor 50 gravide med BMI > 30 kg/m^2^ blev randomiseret til intensiv kostvejledning, har dog fundet, at vægtstigningen kunne holdes på 6 kg, uden negativ påvirkning af det nyfødte barn samtidig med en ikke-statistisk tendens til færre komplikationer for moderen.

Graviditet og fysisk aktivitet:

I Sundhedsstyrelsens anbefalinger fra 2005 tilrådes mindst 30 minutters fysisk aktivitet dagligt til alle kvinder med en normal graviditet, idet fysisk aktivitet under graviditeten giver mindsket risiko for fødselskomplikationer (16), sectio og instrumentel forløsning. En prospektiv undersøgelse af 909 kvinder som var fysisk aktive både før og under graviditeten viste en 69% lavere forekomst af GDM i den mest aktive gruppe (17). Et dansk case-kontrol studie (18) omfattende 201 kvinder med præeklampsi viste at fysisk aktivitet de første 20 uger af graviditeten kunne medføre 35% reduceret risiko for at udvikle præeklampsi.

Da kvinder med højt BMI har betydelig øget risiko for GDM og præeklampsi samt øget risiko for fødselskomplikationer, synes det særligt oplagt at anbefale fysisk aktivitet til denne gruppe kvinder.

Pedometer (skridttæller):

Det har vist sig at være svært at opnå høj deltagelse og kompliance i studier, hvor de gravide randomiseres til motionsprogram. Monitorering med pedometer er effektiv (19,20), men er ikke tidligere undersøgt hos gravide. Pedometer-aktivitet korrelerer med den daglige fysiske aktivitet i opgjort ved selvrapporteret dagbog. (21,22)

I et studie har man monitoreret normalvægtige gravide med accelerometer og fundet at den fysiske aktivitet falder med ca. 10-15% fra GA: 25 uger til GA: 38 uger. (22)

I et tværsnitsstudium har vi målt pedometer-aktivitet hos normalvægtige og overvægtige danske gravide og har fundet at gravide går signifikant flere skridt på hverdage end på weekenddage (7982 skridt/dag vs. 6915 skridt/dag) p< 0,0001. Der er en lille tendens til, at normalvægtige går flere skridt dagligt end overvægtige, men dette er ikke signifikant. De normalvægtige går dog signifikant flere skridt end de overvægtige på weekenddage (7306 skridt/dag vs. 6025 skridt/ dag) p<0,05.

Gravide med BMI≥30 går gennemsnitlig 7135 skridt dagligt. I dette studie vil de gravide som får intervention i form af øget fysisk aktivitet få udleveret et pedometer. De vil blive opfordret til at opnå et dagligt skridttal på 11.000 skridt sv.t. ca. 150% af normal aktivitet.

Diæt:

En nyere undersøgelse, hvor 322 overvægtige randomiseres til ”Middelhavs-kost”, ”Lav-kulhydrat diæt” og ”Lav-fedt diæt” viser, at ”Middelhavs-kost” giver effektivt vægttab samt nedsætter fasteblodsukker og insulin-niveau hos diabetikere (23). Et norsk randomiseret studie af 290 gravide viser, at Middelhavskost nedsætter risiko for præterm fødsel (24).

Behandlingsprogram for overvægtige gravide med BMI>30 hos klinisk diætist svarende til ”Middelhavs-kost”, med udgangspunkt i de 8 kostråd er vedlagt som Bilag 3. I samtalerne tilstræbes afdækning af årsag til overvægt, motivering samt vejledning i korrekt sammensat hypokalorisk kost.

**MATERIALE OG METODER:**

A. Randomiseret interventionsstudium. Effekt af fysisk aktivitet målt ved anvendelse af pedometer, samt intensiveret diæt-intervention hos svært overvægtige gravide.

Hypotese:

Fysisk aktivitet og/eller intensiveret diæt-intervention mindsker den maternelle vægtøgning i graviditeten hos den adipøse gravide samt mindsker risikoen for:

1. patologisk glukosebelastning i graviditeten og dermed risikoen for GDM.

2. makrosomi.

3. nedsætter risikoen for sektio pga. dystoci.

**Inklusionskriterier:** BMI ≥30 kg/m².

Normal graviditet ved nakkefolsdsscanning. Informeret samtykke.

**Eksklusionskriterier:** Under 18 år. Ikke dansktalende. Betydende sygdom hos mater eller foster.

Misbrugsproblematik.

**Inklusionsperiode**: 18 måneder hvor der på Hvidovre fødes ca. 600 børn af mødre med prægravid BMI ≥ 30 kg/m². Med en forventet deltagerprocent på 70 forventes der således inkluderet ca. 420.

Alle overvægtige gravide med BMI≥30 tilbydes flg. Standardregime:

**Standard regime:**

- Klinisk diætist: en generel samtale om kostråd og detaljeret kostanamnese (Se nedenfor)
- Anbefaling om fysisk aktivitet, f.eks. rask gang 30 minutter dgl.
- Tilbydes samtale med anæstesilæge om fødselsforløb, epiduralanlæggelse etc.
- Screening for GDM ved OGTT i uge 13-20 og 27-30
- Udover nakkefoldsskanning ved GA 11-13 og misdannelsesskanning ved GA 18-22 foretages UL bestemmelse af fostervægt i GA 37-38
- Fødsel ifølge afdelingens instruks.

Indhold i initial vejledning ved klinisk diætist:

- Vejning, grundig kostanamnese ved udfyldelse af kostanamneseskema (se bilag 3), adfærdsmønstre i relation til diæt og motion forsøges afdækket. De største og vigtigste problemer behandles i den initiale fase.
- Individuel diætvejledning i en hypokalorisk, fedtfattig kost på 5000-7000 kJ, svarende til ”Middelhavs-kost”, med udgangspunkt i de 8 kostråd og en sund

og varieret kost. Der udleveres skriftligt materiale.

Oversigt over studiedesign:

|  | Kostanamnese | Blodprøver | Andet |
| --- | --- | --- | --- |
| Inklusion / randomisering | X |  |  |
| 13-20 |  | OGTT + projektprøver |  |
| 27-30 |  | OGTT + projektprøver |  |
| 37-38 | X |  | ULS |
| Fødsel |  |  | Navlesnorsprøve |
| 8 uger pp |  |  | Barnets højde og vægt  Amning |
| 1 år pp |  | OGTT + projektprøver | Barnets højde og vægt |

**Metode:**

Alle inkluderede overvægtige gravide bliver, efter første besøg hos diætist, hvor der optages en detaljeret kostanamnese telefon-randomiseret 1:1:1, til interventionsgruppe 1, 2 eller til almindeligt standard regime for alle overvægtige som anført ovenfor.

**Mål med interventionen:** Vægtøgning: <5 kg

**Interventionsgruppe 1:**

- Modtager opfølgende diætist-rådgivning ved skiftevis fremmøde og telefonisk kontakt hver 2. uge.
- Medgives pedometer og opfordres til et gennemsnitligt 11.000 skridt dagligt. Dette svarer til 150 % af gennemsnittet for gravide,hvilket er fundet i et forudgående projekt. Aktiviteten monitoreres 7 dage i træk hver 4. uge.
- Følger i øvrigt det normale svangre program samt øvrige dele af standard regime

**Interventionsgruppe 2:**

- Som interventionsgruppe 1, men uden intensiv opfølgning ved diætist.

**Inklusion og indhentning af samtykke:**

Alle gravide med pre-gravid BMI ≥30 kg/m² indkaldes pr brev til diætist efter modtagelsen af svangrepapirerne hos den visiterende jordemoder på Hvidovre Hospital fra den praktiserende læge. Til alle gravide med BMI≥30 kg/m² som opfylder inklusions- og ikke eksklusionskriterierne vedlægges:

- Skriftlig patientinformation om projektet (bilag 4),
- Folderen: ” Dine rettigheder som forsøgsperson i et biomedicinsk projekt”
- Anamneseskema (bilag 1a).

Ved fremmøde tilbydes patienten:

- Samtale med diætist sv.t. standardregime. Kostanamneseskemaet (bilag 1b) anvendes til alle som diætistens arbejdsredskab.
- Umiddelbart herefter samtale med projektansvarlige mhp mundtlig information om projektet og indhentning af informeret samtykke (underskrift af samtykkeerklæring) i separat lokale. Hvis patienten ønsker informationssamtale en anden dag mhp. at have en bisidder med planlægges dette. Hvis patienten ønsker betænkningstid efter informationssamtalen planlægges nyt møde med projektansvarlige, en anden dag eller senere samme dag.
- Efter informationssamtale og skriftligt samtykke: Randomisering til interventionsgruppe 1, interventionsgruppe 2 eller standardregime (telefon-randomisering).

Gravide med BMI≥30 kg/m² kan herefter inddeles i flg. 5 grupper:

1. Interventionsgruppe 1: Pedometer + intensiv diætvejledning.
2. Interventionsgruppe 2: Pedometer.
3. Inkluderede gravide som følger vanligt svangreprogram samt standardregime.
4. Gravide som møder til samtale hos diætist, men **ikke** ønsker at deltage i projektet.
5. Gravide som udebliver fra indkaldelse til diætist og ikke har fået mundtlig information om projektet.

**Procedure for de 5 grupper gravide:**

1. Interventionsgruppe 1:
   - Efter fremmøde hos diætist sikrer projektansvarlige sig at anamneseskemaet er udfyldt og gennemgår evt. dette med den gravide.
   - Projektansvarlige informerer om diætist-opfølgningsprogram.
   - Projektansvarlige udleverer pedometer og opfordrer til at gå 11.000 skridt dagligt. Sammen med pedometer udleveres: 7 stk. Ugeskema for registrering af skridttal (Bilag 2) og 7 svarkuverter, således at uge-skemaer tilbagesendes løbende. Inkluderede måler fysisk aktivitet i en uge, hver 4. uge gennem hele graviditeten (ca. GA 14,18, 22, 26, 30, 34, 38) På dette skema påføres også aktuel maternel vægt. Første ”skridttælleruge” påbegyndes så tidligt som muligt fra GA 14+0. Sms-kontakt fra projektansvarlige dagen før start af hver registrerings uge tilbydes. Der indhentes skriftlig tilladelse til dette (Bilag 6)
   - Maternel vægt måles.
   - Der planlægges UL ved projektansvarlige i GA 37-38.
2. Interventionsgruppe 2:
   - Efter fremmøde hos diætist sikrer projektansvarlige sig at anamneseskemaet er udfyldt og gennemgår evt. dette med den gravide.
   - Projektansvarlige udleverer pedometer og opfordrer til at gå 11.000 skridt dagligt. Sammen med pedometer udleveres: 7 stk. Ugeskema for registrering af skridttal (Bilag 2) og 7 svarkuverter, således at uge-skemaer tilbagesendes løbende. Inkluderede måler fysisk aktivitet i en uge, hver 4. uge gennem hele graviditeten (ca. GA 14,18, 22, 26, 30, 34, 38) På dette skema påføres også aktuel maternel vægt. Første ”skridttælleruge” påbegyndes så tidligt som muligt fra GA 14+0. Sms-kontakt fra projektansvarlig dagen før start af hver registrerings uge tilbydes. Der indhentes skriftlig tilladelse til dette (Bilag 6)
   - Maternel vægt måles.
   - Der planlægges UL ved projektansvarlige i GA 37-38.
3. Inkluderede gravide som følger vanligt svangreprogram samt standardregime:

- Efter fremmøde hos diætist sikrer projektansvarlige sig at anamneseskemaet er udfyldt og gennemgår evt. dette med den gravide.
- Den gravide følger fremover vanligt svangreprogram samt standardregime.
- Der planlægges UL ved projektansvarlige i GA 37-38.

1. Gravide som møder til samtale hos diætist, men **ikke** ønsker at deltage i projektet:

- Efter fremmøde hos diætist beder projektansvarlige om tilladelse til at anamneseskemaet udfyldes hvis det ikke er gjort og gennemgår evt. dette.
- Den gravide følger fremover vanligt svangreprogram samt standardregime.

1. Gravide som udebliver fra indkaldelse til diætist og ikke har fået mundtlig information om projektet:

- Den gravide møder i henhold til standardregime til lægesamtale i forbindelse med misdannelsscanning ved GA 18-22. Anamneseskemaet efterspørges. Hvis dette ikke er udfyldt udleveres det evt. igen, og den gravide bedes om at udfylde det.

**Projektblodprøver:** I alle tre grupper foretages oral glukose tolerance test (OGTT) der som led i standardregimet tages i 13.-20. og 27.- 30. uge. I forbindelse hermed vil der desuden blive udtaget blod til bestemmelse af c-peptid, CRP og lipid profil, dvs. triglycerider, HDL, LDL, og total cholesterol. Der vil blive oprettet en forsknings-biobank (20 ml blod pr blodprøvetagning) med henblik på senere bestemmelser af blandt andet IL-18, IL-6, TNF alfa.

Blodprøverne vil blive opbevaret i 10 år.

Materialet vil udelukkende blive brugt til analyser mhp afklaring af forhold vdr gravdiditet, motion, diæt og overvægt.

Plasmaglucose og OGTT måles løbende på biokemisk afdeling ved rutinemetoder.

Ved OGTT møder patienten fastende og indtager 75 g glucose. Plasma glucose bestemmes initialt og efter 2 timer.

Ekstraglas til måling af markører for glucose omsætning centrifugeres, af pipetteres og opbevares ved –80 grader til senere analyse.

**Blodprøve fra navlesnor** tages af jdm umiddelbart efter fødslen og efter afnavling af barnet**.** Hvis det er muligt, tages op til 20 ml blod, ligeledes mhp. opbevaring i forskningsbiobank.

**Ultralydsscanning ved GA 37-38: V**ægtestimering og måling af fostervandsmængde. For de inkluderede patienter i gruppe 1,2 og 3 foretages dette af projektansvarlige. I forbindelse med dette gentages udfyldelse af kostanamneseskemaet (se nedenfor), og forsøgspersonen vejes.

**Kliniske data:**

Almindelige baggrundsdata hentes fra journal, og supplerende data indhentes fra anamneseskema, se bilag 1 a+b. Data om svangrekomplikationer, fødsel etc. indhentes fra journal, vandrejournal og fra obstetrisk database. Den sidst registrerede maternelle vægt fra svangrejournal sættes som vægt ved graviditetens afslutning.

**Data ved opfølgning:**

Hvis forsøgspersonerne har givet tilladelse til opfølgning, vil de blive kontaktet pr brev hhv 8 uger efter fødslen og igen i forbindelse med indkaldelse til opfølgende OGTT 1 år efter fødslen med kortfattet spørgeskema vdr barnets højde og vægt, samt oplysning om amning. (Bilag 7)

**Primære endpoints:**

Maternel vægtøgning

Fødselsvægt – i forhold til GA

Fødselsmåde: ukompliceret vaginal fødsel / instrumentel forløsning / sectio

**Sekundære endpoints:**

2 timers blodsukker ved OGTT

Lipidprofil

Forekomst af hypertension eller præeklampsi

Igangsættelse

Behov for vestimulation

Hypoglykæmi hos barnet

Placentas vægt

Aming

**B.** **Spørgeskemaundersøgelse: Belysning af kostvaner/kostændringer hos overvægtige gravide med BMI≥30 kg/m², med hhv. høj og lav vægtøgning i graviditeten.**

**Hypotese:**

Indtagelse af ”Middelhavskost”

- medfører reduktion af vægtøgningen i graviditeten.
- mindsker risikoen for patologisk glukosebelastning i graviditeten og dermed risiko for GDM.
- nedsætter risiko for makrosomi (FV>4000g)
- nedsætter risiko for præterm fødsel.

**Metode:**

Detaljeret kostanamnese (360 spørgsmål) optages først og sidst i graviditeten ved hjælp af spørgeskema anvendt og valideret i undersøgelsen: Bedre sundhed for mor og barn. Betydningen af ændringer i kostens delelementer for maternel vægtøgning, fostervægt, graviditetslængde og graviditetskomplikationer analyseres.

Indlæsning samt analyse af data vil blive foretaget af i samarbejde med statistiker på Statens

Seruminstitut.

**Styrkeberegning ved delprojekt B:** Ved en forventet middelværdi af kolesterol på 270 mg/dl i 37. uge med standard deviation på 40 mg/dl (25) kræves der 129 kvinder i hver gruppe for at detektere en 5 % reduktion af kolesterol (alfa = 0,05: beta = 0.20). Dette antal kan med en styrke på 65 % og alfa på 0.05 finde en 50% stigning i graviditetskomplikationer (forudsætning 25 % graviditetskomplikationer.) Vi stiler mod 140 i hver gruppe for at tage højde for e.v.t. dropouts, dvs ialt 420 kvinder.

**DATABEHANDLING/ STATISTIK:**

Undersøgelsen er randomiseret

Data vil blive analyseret i samarbejde med statistiker Søren Lundby Christensen, Cand. Scient. Ph.d. Institut for sundhedsvidenskab og teknologi. Ålborg Universitet.

Sammenhæng mellem kalorierestriktion, fysisk aktivitet og den maternelle vægtstiging opgøres efter stratificering efter initial maternel vægt.

Indlæsning samt analyse af data fra kostanamneseskemaerne vil blive foretaget af i samarbejde med statistiker på Statens Seruminstitut.

**RISICI, BIVIRKNINGER OG ULEMPER:**

Der er umiddelbart ingen risiko eller bivirkninger for forsøgspersonerne i forbindelse med projektet. Eneste ulempe er det lille tidsforbrug i forbindelse med aflæsning og registrering af skridttal. Desuden er der for de gravide som randomiseres til opfølgning ved diætist ulempen ved tidsforbruget på dette (15 min. Telefonsamtale hver 14. dag, 15. minutters konsultation ved fremmøde hver 4. uge). De blodprøver, som tages i forbindelse med projektet, tages samtidigt med at den gravide møder til OGTT og alligevel får taget blodprøve. Hun skal således ikke møde op særligt mhp projekt-prøver og skal ikke stikkes flere gange end de kvinder som ikke deltager i projektet under graviditeten. Hvis hun accepterer opfølning vil der dog et år efter fødslen laves en ekstra oral glukose tolerance test mhp undersøgelse for sukkersyge.

**ETISKE OVERVEJELSER:**

Som nævnt ovenfor er der ingen risiko eller bivirkninger forbundet med deltagelse i projektet. Det forventes, at de kvinder, som randomiseres til intervention, får nedsat risiko for at udvikle graviditets- og fødselskomplikationer, samt at resultaterne kan få betydning for fremtidig behandling af overvægtige gravide.

Positive såvel som negative resultater vil blive offentliggjort i et videnskabeligt tidsskrift.

Der vil blive foretaget anmeldelse af projektet til Datatilsynet samt til De Videnskabsetiske Komiteer.

**ØKONOMI:**

Der er bevilliget 300.000 kr. til projektet fra Sygekassernes helsefond. Der vil søges fondsmidler fra flere fonde. Hverken forsøgsansvarlige eller andre i projektgruppen har økonomisk tilknytning til Sygekassernes Helsefond, eller til andre eventuelle støttegivere.

Der gives ikke vederlag til forsøgspersonerene.

**BILAG:**

1. a. Anamneseskema inklusiv skema om motionsvaner.

b. Særligt kostananmneseskema

1. Dataark for pedometer mhp registrering af skridttal.

1. Behandlingsprogram for overvægtige gravide med BMI>30 hos klinisk diætist
2. Skriftlig patientinformation om projektet.

1. a. Samtykkeerklæring.

b. Samtykkeerklæring til indehavere af forældremyndighed mhp. udtagelse af blod fra navlesnoren.

1. Blanket til informeret samtykke om kontakt pr. SMS til gravide i interventionsgrupperne.
2. Spørgeskema mhp opfølgning 8 uger og 1 år post partum

**Referencer:**

1. Heitmann BL. Ten-year trends in overweight and obesity among Danish men and women aged 30 – 60 years. Int J Obes 2000; 24: 1347-52.
2. Solomon CG, Willett WC, Carey VJ, Rich-Edwards J, Hunter DJ, Colditz GA et al. A prospective study of pregravid determinants of gestational diabetes mellitus. JAMA 1997; 278: 1078-83.
3. Edwards LE, Hellerstedt WL, Alton IR, Story M, Himes JH. Pregnancy complications and birth outcomes in obese and normal-weight women: effects of gestational weight change. Obstet Gynecol 1996; 87: 389-94.
4. O`Brien TE, Ray JG, Chan W. Maternal body mass index and the risk of pre-eclampsia: a systematic overview. Epidemiology 2003; 14: 368-74.
5. Sebire NJ, Jolly M, Harris JP, Wadsworth J, Joffe M, Beard RW et al. Maternal obesity and pregnancy outcome: a study of 287 213 pregnancies in London. Int J of Obes. 2001; 25: 1175-82.
6. Brost BC, Goldenberg RL, Mercer BM, Iams JD, Meis PJ, Moawad AH et al. The preterm prediction study: association of cesarean delivery with increases in maternal weight and body mass index. Am J Obstet Gynecol 1997; 177:333-41.
7. Perlow JH, Morgan MA. Massive maternal obesity and perioperative cesarean morbidity. Am J Obstet Gynecol 1994; 170: 560-5.
8. Werler MM, Louik C, Shapiro S, Mitchell AA. Pre-pregnant weight in relation to risk of neural tube defects. JAMA 1996; 275: 1089-92
9. Baeten JM, Bukusi EA, Lambe M. Pregnancy complications and outcomes among overweight and obese nulliparous women. Am J Public Health 2001; 91: 436-40.
10. Kristensen J, Vestergaard M, Wisborg K,Kesmodel U, Secher NJ. Pre-pregnancy weight and the risk of stillbirth and neonatal death. BJORG 2005 ;112(4):403-8.
11. Jensen DM, Ovesen P, Beck-Nielsen H, Mølsted-Pedersen L, Sørensen B, Vinter C et al. Gestational weight gain and pregnancy outcomes in 481 obese glucose-tolerant women. Diabetes Care 2005; 28(9): 2118-22.
12. Cedergren MI. Optimal Gestational Weight Gain for body Mass Index Categories. Obstet Gynecol 2007;110:759-64.
13. Kiel DW, Dodson EA, Artal R, Boehmer TK, Leet T. Gestational Weight Gain and Pregnancy Outcomes in Obese Women. Obstet Gynecol 2007;110:752-8.
14. Olsen SF, Dragsted LO, Hansen HS, Michaelsen KF, Milman N, Ovesen L et all. Kost til gravide, det videnskabelige grundlag for råd om kost i forbindelse med graviditeten. En rapport fra Ernæringsrådet 2005: 19-23.
15. Wolff S, Toubro S, Vangsgaard K, Legarth J, Astrup A. Limitation of gestational weight gain in obese - a randomised controlled trial. Int J Obesity 2008;32,495-501.
16. Clapp JF, III, The course of labor after endurance exercise during pregnancy. Am J Obstet Gynecol 1990;163:1799-1805
17. Dempsey JC, Soerensen TK,Williams MA et al. Prospective study of gestational diabetes mellitus risk in relation to maternal recreational physical activity before and during pregnancy. Am J epidemiol 2004;159:663-70
18. Soerensen TK, Willliams MA, Lee IM et al. Recreational physical activity during pregnancy and risk of pre-eclampsia. Am J. Hypertension2003;41:1273-80
19. Speck BJ, Looney SW. Effects of minimal intervention to increase physical activity in women: daily activity records. Nurs Res. 2001 Nov-Dec; 50(6):374-8.
20. Araiza P, Hewes H, Gashetewa C, Vella CA, Burge MR. Efficacy of pedometer-based physical activity program on parameters of diabetes control in type 2 diabetes mellitus. Metabolism. 2006 Oct;55(10):1382-7.
21. Bjorgass M, Vik JT, Saeterhaug A, Langlo L, Sakshaug T, Moohus RM, Grill V. Relationship between pendometer-regestered activity, aerobic capacity and self-reported activity and fitness in patient with type 2 diabetes. Diabetes Obes Metab. 2005, (6): 737-44
22. Lindseth G, Vari P. Measuring Physical Activity during pregnancy. Western Journal of nursing Research 2005; 27 (6): 722-34
23. Shai I, Schwarzfuchs D et al. Weight Loss with a Low-Carbohydrate, Mediterranean, or Low-Fat Diet. N Engl J Med 2008;359:229-41.
24. Khoury, J, Henriksen T et al. Effect of cholesterol-lowering diet on maternal cord, and neonatal lipids, and pregnancy-outcome: a randomized clinical trial. Am J Obstet Gynecol 2005;193,1292-301.
25. Gørbitz, C Bergei CS, Sivertsen M, Olse L. Diet and lipid status in pregnant women. Tidsskr. Nor Lægeforen 1995;115:1358-60.

**LÆGMANDSRESUMÉ:**

**Effekt af livsstilsintervention i form af diæt og motion samt belysning af kostvaners og biomakørers (markører i blodet) betydning for graviditets- og fødselskomplikationer hos overvægtige gravide:**

**Baggrund:**

Overvægt er et stigende problem i Danmark. Overvægtige gravide har en øget risiko for forekomst af en lang række komplikationer i graviditeten såsom graviditetsbetinget sukkersyge, forhøjet blodtryk samt svangerskabsforgiftning (Tilstand, som kan opstå i graviditeten med forhøjet blodtryk, udskillelse af æggehvidestoffer i urinen, hævede hænder og fødder og evt. hævelse i ansigtet) . Et højt BMI øger risikoen for, at fødslen igangsættes samt for kejsersnit og sygelighed i forbindelse med operation.

Kvinder, som udvikler graviditetsbetinget sukkersyge, har stor risiko for at udvikle sukkersyge senere i livet. Det er derfor relevant at udvikle effektiv livsstilsintervention (forsøg på påvirkning til sundere livsstil) i graviditeten. Livsstilsændring i graviditeten forventes at øge sundheden og nedsætte risiko for fedme og sukkersyge for børnene i den næste generation.

Den undersøgte interventionsform med skridttæller alene indebærer et samfundsmæssigt lavt ressourceforbrug og kan let implementeres i det kliniske arbejde. Skridttællerern har i andre studier vist sig at være et effektivt redskab til opnåelse af bl.a. vægttab.

**Formål:**

Undersøgelsens formål er at belyse effekten af fysisk aktivitet målt ved anvendelse af skridttæller, samt af diæt-intervention hos svært overvægtige gravide med BMI ≥30 kg/m². Vi vil undersøge, om øgning af den fysiske aktivitet under anvendelse af skridttæller kan nedsætte den gravides vægtøgning samt nedsætte risiko for graviditets- og fødselskomplikationer.

Samtidig undersøges effekten af diætvejledning ved diætist og opfølgning herpå hver 14. dag.

Desuden foretages detaljeret kostregistrering mhp. efterfølgende analyse af kostens betydning for vægtøgning i graviditeten og graviditets komplikationer.

**Forsøgsdeltagere:**

Antal: 420

Inklusion: BMI ≥30 kg/m². Normal graviditet ved nakkefolsdsscanning. Informeret samtykke.

Eksklusion: Under 18 år. Ikke dansktalende. Betydende sygdom hos mor eller foster.

Misbrugsproblematik.

**Design:**

A. Randomiseret interventionsstudium. Dvs forsøgsdeltagerne fordeles tilfældigt i 3 grupper. Én gruppe som får intervention i form af opfordring til øget fysisk aktivitet og anvendelse af skridttæller samt opfølgning af diætist. En anden gruppe som får intervention i form af opfordring til øget fysisk aktivitet og anvendelse af skridttæller. En tredie gruppe som ikke får intervention, men følger afdelingens standardregime for overvægtge gravide.

B. Spørgeskemaundersøgelse i form af udfyldelse af detaljeret kostanamneseskema.

**Metoder:**

A. Forsøgpersonerne fordeles tilfældigt i 3 grupper som beskrevet ovenfor.

De gravide, som får intervention i form af anvendelse af skridttæller, medgives skridttæller og opfordres til at opnå gennemsnitligt 11.000 skridt dagligt. Dette svarer til 150 % af gennemsnittet for gravide. Sammen med skridttælleren udleveres 7 stk. ugeskema for registrering af skridttal og 7 svarkuverter, således at uge-skemaer tilbagesendes løbende. Aktiviteten monitoreres 7 dage i træk hver 4. uge. gennem hele graviditeten. På dette skema påføres også aktuel maternel vægt.

De gravide, som får intervention i form af opfølgning af diætist modtager udover standardvejledning om en hypokalorisk, fedtfattig kost, svarende til ”Middelhavs-kost”, opfølgende diætist-rådgivning ved skiftevis fremmøde og telefonisk kontakt hver 2. uge.

Mhp undersøgelse for sukkersyge foretages som led i standardregimet en ”sukkerbelastnings-test” tidlig og sent i graviditeten samt 1 år efter fødslen. (Test hvor man møder fastende og indtager 75 g sukker. Der tages blodprøve initialt og efter 2 timer) I forbindelse med hver af disse 3 prøver tages tages 20 ml ekstra blod som opbevares i 10 år i en forskningsbiobank til senere brug. Desuden tages efter fødslen og afnavling af barnet op til 20 ml blod fra navlesnoren, ligeledes mhp. opbevaring i forskningsbiobanken. Materialet vil blive brugt til analyser mhp afklaring af forhold vdr gravdiditet, motion, diæt og overvægt.

B. Først og sidst i graviditeten udfylder alle et kostregistreringsskema (360 spørgsmål), som har været anvendt og valideret i undersøgelsen "Bedre sundhed for mor og barn". Ved hjælp af dette analyseres kostens betydning for vægtøgning i graviditeten og graviditets komplikationer.

**Inkludering og indhentning af informeret samtykke:**

Som led i afdelingens standardregime indkaldes alle gravide med BMI≥30 kg/m² til vejledende samtale med diætist så tidligt som muligt i graviditeten.Til de som opfylder inklusions- og ikke eksklusionskriterierne fremsendes med indkaldelsen den skriftlige information om projektet.

Umiddelbart efter samtalen med diætisten informerer projektansvarlige mundtligt om projektet i et andet lokale. Hvis den gravide ønsker informationssamtale en anden dag mhp. at have en bisidder med planlægges dette. Hvis hun ønsker betænkningstid efter informationssamtalen planlægges nyt møde med projektansvarlige, enten senere samme dag eller en anden dag.

På samtykkeerklæringen anmodes særskilt om tilladese til opbevaring af blodprøve i forskningsbiobank, samt om tilladelse til at der foretages opfølgning 8 uger og 1 år efter fødslen.

Efter informationssamtale og skriftligt samtykke (underskrivning af samtykkeerklæring) randomiseres til interventionsgruppe 1, interventionsgruppe 2 eller standardregime ved telefon-randomisering. Hvis forsøgsdeltageren randomiseres til én af de to interventionsgrupper gives herefter mere detaljeret vejledning om det praktiske forløb og registreringsskemaer til skridttællermonitorering og svarkuverter udleveres.

**Videnskabsetik:**

Der er umiddelbart ingen risiko eller bivirkninger for forsøgspersonerne i forbindelse med projektet. Eneste ulempe er det lille tidsforbrug i forbindelse med aflæsning og registrering af skridttal. Desuden er der for de gravide som randomiseres til opfølgning ved diætist ulempen ved tidsforbruget på dette (15 min. Telefonsamtale hver 14. dag, 15. minutters konsultation ved fremmøde hver 4. uge). De blodprøver som tages i forbindelse med projektet tages samtidigt med at den gravide møder til ”sukkerbelastningstest” og alligevel får taget blodprøve. Hun skal således ikke møde op særligt mhp projekt-prøver og skal ikke stikkes flere gange end de kvinder, som ikke deltager i projektet under graviditeten. Hvis hun accepterer opfølning, vil der dog et år efter fødslen laves en ekstra ”sukkerbelastningstest” mhp undersøgelse for sukkersyge.

Det forventes at de kvinder som randomiseres til intervention får nedsat risiko for at udvikle graviditets- og fødselskomplikationer, samt at resultaterne kan få betydning for fremtidig behandling af overvægtige gravide.

**Økonomi:**

Der er bevilliget 300.000 kr. til projektet fra Sygekassernes helsefond. Der vil søges fondsmidler fra flere fonde. Hverken forsøgsansvarlige eller andre i projektgruppen har økonomisk tilknytning til Sygekassernes Helsefond, eller til andre eventuelle støttegivere.

Der gives ikke vederlag til forsøgspersonerene.
