# Supplementary material for: Intake of Sweets, Snacks and Soft Drinks Predicts Weight Gain in Obese Pregnant Women: Detailed Analysis of the Results of a Randomised Controlled Trial
Source: PLoS One. 2015 Jul 20;10(7):e0133041. doi: 10.1371/journal.pone.0133041 (PMC4507874; doi:10.1371/journal.pone.0133041)
Supplement: S4 File — (DOC) [file pone.0133041.s004.doc]

**A randomised controlled trial of the effect of dietary and/or physical**

**activity intervention in obese pregnant women**

PROJECT GROUP

Departmental Physician Kristina Renault ^1^

Consultant Kirsten Riis Andreasen ^1^

Consultant Lisbeth Nilas ^1^

Professor, Niels Jørgen Secher ^1^

Consultant Maiken Lundstrøm ^1^

Consultant Kirsten Nørgaard ^2^

Clinical Dietician Anette Martinsen ^2^

Senior Research, Professor Sjurdur F. Olsen ^3^

1: Gynaecologic Obstetric Department

2: Endocrinologic Department

Hvidovre Hospital

Kettegårds Allé 30

2650 Hvidovre

3: Department for Epidemiologic Research

Statens Serum Institut,

Artillerivej 5

2300 Copenhagen S

PROJECT LEADER

Kristina Renault

Translation from Danish by:

Associate Professor, MD., DMSci.

Edwin Stanton Spencer

Århus University Hospital,

Denmark.

The following projects are planned.

1. **Randomised intervention study: Effect of physical activity measured by pedometer and intensive dietary intervention in obese pregnant women with BMI≥30 kg/m².**

A randomised intervention study comparing standard dietary advice regimen with a recommendation of increased physical activity monitored by pedometer together with and without intensive dietary advice based on a Mediterranean diet. Intensive dietary advice given by a dietician and checked ever other week alternately by personal appearance or telephone consultation

**PURPOSE:**

- To illustrate the effect of intervention on activity level, diet, weight gain, glucose tolerance, lipid profile in test subjects and clinical parameters in the child.
- To illustrate factors that influence participation and compliance in connection with life-style intervention.

1. **Questionnaire study. Investigate dietary habits/dietary changes in obese pregnant women with BMI≥30 kg/m², with great and low weight gain during pregnancy:**

Detailed dietary history (360 questions) taken at the beginning and end of pregnancy as was used and validation in the study: “Better health for mother and child”

**PURPOSE:**

- To analyse the importance of changes in elements of the diet for maternal weight gain, length of pregnancy and complications of pregnancy.

**MATERIALS AND METHODS**.

1. **Randomised intervention study. Effect of physical activity measure with use of a pedometer together with dietary intervention in heavily obese pregnant women.**

Hypothesis:

Physical activity and or intensive dietary intervention will lessen material weigh gain during pregnancy in obese pregnant women and lessen the risk of

1. pathologic glucose tolerance during pregnancy and thereby the risk of GDM.

2. macrosomia.

3. reduce risk of caesarean section because of dystocia.

**Inclusion criteria:** BMI ≥30 kg/m².

Normal pregnancy with normal neck fold scan. Informed consent.

**Exclusion criteria:** Under 18 years. Non-Danish speaker. Serious disease in mother or fetus. Alcohol or drug dependency.

**Inclusion period**: 18 months during which 600 children are born at Hvidovre Hospital of mothers with a pre-pregnancy BMI ≥ 30 kg/m². With an expected participation percentage of 70, we expect to include 420 in the study.

In accordance with the standard regimen, all obese pregnancy women with BMI≥30 are offered:

**Standard regimen:**

- Clinical dietician: a general consultation with dietary advice and a detailed dietary history. (see below)
- Recommendation regarding physical activity, for instance rapid walking 30 minutes/day.
- Offered consultation with anaesthesiologist regarding course of birth, epidural, etc.
- Screening for GDM with OGTT in weeks 13-20 and 27-30
- In addition to neck fold scan in GA 11-13 and deformity scanning at GA 18-22 US (ultrasound) determination of fetal weight at GA 37-38
- Delivery according to departmental instructions.

Content of initial advice by dietician:

- Weight, detailed dietary history with completion of a diet form (see appendix 3), attempt to determine behavioural patterns in relation to diet and physical activity. The largest and most importance problems are dealt with in the initial phase.
- Individual dietary advice on hypocaloric, low fat diet of 5000–7000 kJ, corresponding to “Mediterranean diet”, based on the 8 dietary suggestions and a healthy and varied diet. Written information handed out.

**Overview of study d**esign:

|  | Dietary history | Blood tests | Other |
| --- | --- | --- | --- |
| Inclusion / randomisation | X |  |  |
| 13-20 |  | OGTT + project tests |  |
| 27-30 |  | OGTT + project tests |  |
| 37-38 | X |  | US |
| Birth |  |  | Cord Blood Tests |
| 8 weeks pp |  |  | Child’s height and weight  Breastfeeding |
| 1 year pp |  | OGTT + project tests | Childs height and weight |

**Method:**

After the first visit to the dietician during which a detailed dietary history is taken, all included obese pregnant women will be telephone randomised 1:1:1, to intervention groups 1, 2 or to general standard regimen for all obese pregnant women as given above.

**Intervention aim:** Weight gain: <5 kg

**Intervention group 1:**

- Receive follow-up dietary advice by alternatively personal or telephone contact every 2^nd^ week. Given pedometer and advised to take 11,000 steps/day. This corresponds to 150 % of average for pregnant women, determined in previous project. Activity monitored for 7 consecutive days every 4th week.
- Follows in addition the normal pregnancy program and the other aspects of the standard regimen.

**Intervention group 2:**

- As intervention group 1, but without intensive follow-up by dietician.

**Inclusion and obtaining consent:**

All pregnant women with pre-pregnancy BMI ≥30 kg/m² will be summoned by letter to the dietician after receipt of notice of pregnancy by the visiting midwife at Hvidovre Hospital from the GP. To all pregnant women with BMI≥30 kg/m² who fulfil the inclusion criteria is sent:

- Written information on the project (appendix 4),
- The folder: “Your rights as a research subject in a biomedical project”
- History form (appendix 1a).

At time of visit the patient is offered:

- Consultation with dietician according to standard regimen. Dietary history form (appendix 1b) as used by all dieticians.
- Immediately hereafter, consultation with project leader with oral information on the project and receipt of informed consent (signing of consent form) in separate room. If the patient wishes to receive information on the project on another day, for instance, if they want someone to be with them, this is then arranged. If the patient wishes to have time to consider participation after being informed of the project, then a new meeting is planned with the project leader either the same day or another day.
- After the information consultation and written consent: Randomisation to intervention group 1, intervention group 2 or standard regimen (telephone randomisation).

Pregnant women with BMI≥30 kg/m² are then divided into the following 5 groups:

1. Intervention group 1: Pedometer + intensive dietary advice.
2. Intervention group 2: Pedometer.
3. Pregnant women who follow normal pregnancy program and standard regimen.
4. Pregnant women who come to a consultation with the dietician, but who **do not** wish to take part in the project.
5. Pregnant women who do not respond to summons to dietician and who have not received oral information about the project.

**Procedure for the 5 groups of pregnant women:**

1. Intervention group 1:
   - After the consultation with the dietician, the project leader assures that the history form is filled out and goes through it with the pregnant women.
   - Project leader informs about dietician follow-up program.
   - Project leader hands out pedometer and encourages a step count of 11,000 steps a day. Together with the pedometer is given: 7 copies of week-form for registration of step count (Appendix 2) and 7 return envelopes so that the week-forms can be returned on-going. Those included measure physical activity during 1 week every 4^th^ week during the whole pregnancy. (circa GA 14,18, 22, 26, 30, 34, 38) On this form current weight is noted. The first pedometer week beginning as soon as possible from GA 14+0. SMS contact from project leader the day before the start of every registration week is offered. Written permission for this is obtained. (Appendix 6)
   - Maternal weight noted.
   - US planned by project leader at GA 37-38.
2. Intervention group 2:
   - After the consultation with the dietician, the project leader assures that the history form is filled out and goes through it with the pregnant women.
   - Project leader informs about dietician follow-up program.
   - Project leader hands out pedometer and encourages a step count of 11,000 steps a day. Together with the pedometer is given: 7 copies of week-form for registration of step count (Appendix 2) and 7 return envelopes so that the week-forms can be returned on-going. Those included measure physical activity during 1 week every 4^th^ week during the whole pregnancy. (circa GA 14,18, 22, 26, 30, 34, 38) On this form current weight is noted. The first pedometer week beginning as soon as possible from GA 14+0. SMS contact from project leader the day before the start of every registration week is offered. Written permission for this is obtained. (Appendix 6)
   - Maternal weight noted.
   - US planned by project leader at GA 37-38.
3. Pregnant women who follow usual pregnancy programme and standard regimen.

- After consultation with dietician, the project leader assures that the history form is filled out and goes through it if necessary with the pregnant women.
- Then pregnant women follow hereafter the usual pregnancy programme and standard pregnancy regimen.
- US planed by project leader at GA 37-38.

1. Pregnant women who come to consultation with the dietician, but who **do not** want to take part in the project:

- After consultation with dietician the project leader requests that the history form is filled out and goes through it.
- The pregnant woman follows the usual pregnancy programme and standard regimen.

1. Pregnant women who do not respond to summons to the dietician and who have not received oral information about the project:

- The pregnant woman comes to a consultation with a doctor in accordance with the standard regimen in order to arrange a malformation scan at GA 18-22. The history form is asked about. If this has not been filled out, it is given out again and the pregnant woman is asked to fill it out.

**Project blood tests:** In all three groups an oral glucose tolerance test (OGTT) is done as part of the standard regimen at 13–20 and 27–30 weeks. In connection with these tests, blood will be taken for determination of c-peptid, CRP and lipid profile, (triglycerides, HDL, LDL, and total cholesterol) A research biobank will be set up (20 ml blood per blood sampling session) with the aim of later determination of, among others, IL-18, IL-6, TNF alfa.

Blood tests will be stored for 10 years

The material will only be used for analyses regarding determination of conditions related to pregnancy, physical activity, diet and obesity.

Plasma glucose and OGTT measured regularly at the department of biochemistry using routine methods.

For the OGTT the patient is fasting and ingests 75 g glucose. Plasma glucose determined initially and after 2 hours.

Extra tubes for measurement of markers for glucose metabolism are centrifuged, pipetted off and stored at –80 degrees until later analysis.

**Cord Blood samples** are taken immediately after birth and cutting of umbilical cord. If possible up to 20 ml blood is taken, and stored in the research biobank.

**Ultrasound (US) scans at GA 37-38:** Estimation of weight and measurement of amniotic fluid. This is done by project leader in patient groups 1, 2 and 3. In connection with this, the dietary history form is filled out again (see below) and the patient weighed.

**Clinical data:**

General background data from the chart and supplementary data from the history form, see appendix 1 a+b. Data on pregnancy complications, birth, etc., from the chart, midwife notes and the obstetrical database. Maternal weight from pregnancy chart is noted as the weight at the end of pregnancy.

**Data on follow-up:**

If the patient has given permission for follow up, they are contacted by letter 8 weeks after birth and again in connection to summons to follow-up OGTT 1 year after birth and given a short questionnaire regarding the height and weight of the child and information on breast feeding. (Appendix 7)

**Primary endpoints:**

Maternal weight increase

Birth weight – in relation to GA

Method of birth: uncomplicated vaginal birth / instrumental delivery / sectio

**Secondary endpoints:**

2 hour blood glucose with OGTT

Lipid profile

Presence of hypertension or preeclampsia

Induction

Need to promote uterine contractions

Hypoglycaemia in child

Placenta’s weight

Breast feeding

**B.** **Questionnaire study: Elucidation of dietary habits/ diet changes in obese pregnant women med BMI≥30 kg/m², with high or low weight grain during pregnancy.**

**Hypothesis:**

Ingestion of “Mediterranean diet”

- Reduces weight increased during pregnancy.
- Lessens risk of pathologic glucose intolerance in pregnancy and thereby risk of GDM.
- Reduces risk of macrosomia (FV>4000g)
- Reduces risk of pre-term birth.

**Method:**

A detailed dietary history (360 questions) will be taken at the beginning and end of pregnancy using a questionnaire used and validated in the study “Better health for mother and child”. Importance of changes in the individual elements of the diet for the increase in maternal weight, fetal weight, length of pregnancy and pregnancy complications will be analysed.

Collection and analysis of date will be done in association with statisticians at Statens

Seruminstitut (the State Serum Institute).

**Calculation of strength in part-project B:** With an expected middle value for cholesterol of 270 mg/dl in week 37, with a standard deviation of 40 mg/dl (25), 129 women are needed in each group to detect a 5% reduction in cholesterol (alfa = 0.05: beta = 0.20). This number with a strength of 65% and an alfa of 0.05 will determine a 50% increase in pregnancy complications (prerequisite 25% pregnancy complications.) We aim to include 140 in each group in order to allow for any dropouts, **thus a total of 420 women**.

**ETHICAL CONSIDERATIONS:**

There is no risk or side effects associated with participation in the project . It is expected that the women randomised to intervention will have a reduced risk of developing pregnancy and labour complications and that the results can be expected to be important for the future treatment of obese pregnant women.

Positive as well as the negative results will be published in a scientific medical journal.

The project will be reported to Datatilsynet (Danish Data Protection Agency) and the medical ethics committees.

**ECONOMY:**

A sum of 300,000 DKK will be donated to the project by Sygekassernes helsefond. Money will also be sought from several funds. Neither the project leader nor others involved in the project group have any economic relation with Sygekassernes Helsefond, or any other possible contributors.

Participants will receive no economic compensation.
