# Supplementary material for: Intake of Sweets, Snacks and Soft Drinks Predicts Weight Gain in Obese Pregnant Women: Detailed Analysis of the Results of a Randomised Controlled Trial
Source: PLoS One. 2015 Jul 20;10(7):e0133041. doi: 10.1371/journal.pone.0133041 (PMC4507874; doi:10.1371/journal.pone.0133041)
Supplement: S5 File — (PDF) [file pone.0133041.s005.pdf]

Region Hovedstaden

Koncern Sekretariatet

De Videnskabsetiske Komiteer
i Region Hovedstaden

Kongens Vænge 2
3400 Hillerød

Telefon 48 20 50 00
Direkte 48205726
Fax 48 20 57 77
Web www.regionhovedstaden.dk

CVR/SE-nr: 29 19 06 23

Protokol nr.: H-D-2008-119

Journal nr.: H-D-2008-119
Ref.: Lone Gundelach

Dato: 26. januar 2009

Afdelingslæge
Kristina Renault
Hvidovre Hospital
Gynækologisk -Obstetrisk Afdeling
Kettegårds Allé 30
2650 Hvidovre

Effekt af livsstilsintervention i form af diæt og motion, samt belysning af kostvaners og biomarkørers betydning for graviditets- og fødselskomplikationer.

Den 5. januar 2009 har komité D modtaget rettet materiale i henhold til komiteens brev af 17. december 2008.

Projektet er herefter **godkendt** i henhold til lov om et videnskabsetisk komitésystem, lov nr. 402 af 28. maj 2003 med senere ændringer.

Godkendelsen gælder for det anmeldte forsøgssted, den anmeldte forsøgsansvarlige samt for den angivne forsøgsperiode.

Godkendelsen gælder til den 1. marts 2012 og omfatter følgende dokumenter:

Protokol af 28. december 2008
Deltagerinformation af 28. december 2008
Samtykkeerklæringer af 28. december 2008
øvrige dokumenter modtaget den 5. januar 2009

Iværksættelse af projektet i strid med godkendelsen kan straffes med bøde eller fængsel, jf. komitélovens § 29.

Ændringer:

Foretages der væsentlige ændringer i protokolmaterialet under gennemførelsen af projektet, skal disse anmeldes til komiteen i form af tillægsprotokoller. Ændringerne må først iværksættes efter godkendelse fra komitéen, jf. komitélovens § 23, stk. 1, nr. 1.

Anmeldelse af tillægsprotokoller skal ske elektronisk på www.drvk.dk med det allerede tildelte anmeldelsesnummer og adgangskode.

Væsentlige ændringer er bl.a. ændringer, der kan få betydning for forsøgspersonernes sikkerhed, fortolkning af den videnskabelige dokumentation, som projektet bygger på samt gennemførelsen eller ledelsen af projektet. Det kan fx være ændringer i in- og eksklusionskriterier, forsøgsdesign, antal forsøgspersoner, forsøgsprocedurer, behandlingsvarighed, effektparametre, ændringer om de forsøgsansvarlige eller

forsøgssteder samt indholdsmæssige ændringer i det skriftlige informationsmateriale til forsøgspersonerne.

Hvor nye oplysninger betyder, at forskeren overvejer at ændre proceduren eller stoppe forsøget, skal komiteen orienteres om det.

Bivirkninger og hændelser:

Komiteen skal omgående underrettes, hvis der under projektet optræder alvorlige bivirkninger eller alvorlige hændelser, jf. komitélovens § 22, stk. 3.

Én gang årligt i hele forsøgsperioden skal komitéen have tilsendt en liste over alle alvorlige bivirkninger og alvorlige hændelser, som er indtruffet i forsøgsperioden sammen med en rapport om forsøgspersonernes sikkerhed, jf. komitélovens § 22, stk. 4.

Materialet skal være på dansk. Listen over alvorlige bivirkninger og alvorlige hændelser kan dog være på engelsk, hvis der er vedlagt et dansk resumé.

Afslutning:

Den forsøgsansvarlige skal senest 90 dage efter afslutningen af projektet underrette komiteen herom, jf. komitélovens § 22, stk. 5.

Afbrydes projektet tidligere end planlagt, skal en begrundelse herfor sendes til komiteen senest 15 dage efter, at beslutningen er truffet, jf. komitélovens § 22, stk. 5.

Hvis projektet ikke påbegyndes, skal dette samt årsagen hertil meddeles komiteen.

Komiteen beder om kopi af den afsluttende forskningsrapport eller publikation, jf. komitélovens § 22, stk. 2. Vi skal i den forbindelse gøre opmærksom på, at der er pligt til at offentliggøre såvel negative som positive forsøgsresultater, jf. komitélovens § 14, stk. 1, nr. 6.

Tilsyn:

Komiteen fører tilsyn med, at projektet udføres i overensstemmelse med godkendelsen, jf. komitélovens § 22, stk. 1.

Med venlig hilsen

Birgit Tystrup

Birgit Tystrup
Formand
